# An associational study: preschool teachers’ acceptance and self-efficacy towards Educational Robotics in a pre-service teacher training program

**DOI:** 10.1186/s41239-021-00264-z

**Published:** 2021-05-31

**Authors:** Despoina Schina, Cristina Valls-Bautista, Anna Borrull-Riera, Mireia Usart, Vanessa Esteve-González

**Affiliations:** Applied Research Group in Education and Technology (ARGET), University of Rovira i Virgili, Tarragona, Spain

**Keywords:** Educational robotics, Preschool education, Pre-service teachers, Self-efficacy, Teacher acceptance, Teacher training, Teachers’ perceptions

## Abstract

**Purpose:**

This study explores pre-service preschool teachers’ acceptance and self-efficacy towards Educational Robotics (ER) during a university course, and also examines their perceptions of the course.

**Methodology:**

This is a one-group intervention study with an associational research design that includes both quantitative and qualitative research methods: two pre-questionnaires and two post-questionnaires on pre-service teachers’ acceptance and self-efficacy towards ER, and participants’ training journals.

**Findings:**

The results show that pre-service teachers’ acceptance and self-efficacy towards ER improved after they completed the ER teacher training course. There was a significant difference between the start and the end of the ER training in the pre-service teachers’ acceptance of ER in the areas of perceived ease of use, enjoyment and attitudes, and in their self-efficacy. The findings based on the training journals show that participants positively evaluated the course. The participants also provided suggestions for improving it, such as additional training sessions, resources and time for experimentation.

**Value:**

Our study reveals the impact of an ER training program and showcases the importance of integrating ER in pre-service teachers’ education.

## Introduction

Educational robotics (ER) is an educational tool (Frangou et al., 2008) that provides new and extended possibilities for learning (Shin & Kim, 2007). As previous literature has indicated, students can learn robotics, learn by robotics, and learn with robotics (Gaudiello & Zibetti, 2016). Learning robotics refers to students becoming familiarized with technology, engineering, and robotics. ER has many benefits in relation to engineering and programming skills (Barker & Ansorge, 2007; Nugent et al., 2009). Learning by robotics means that learners acquire knowledge of a certain subject through robotics, and thus acquire multidisciplinary benefits in mathematics (Barker & Ansorge, 2007; Hussain et al., 2006; Nugent et al., 2009), science (Barker & Ansorge, 2007) and other disciplines. Students learn with robotics when the learning and teaching process is supported by humanized robots that act as assistants. Integrating ER into the school curriculum should be promoted given that it benefits students’ learning across multiple disciplines, and facilitates the acquisition of twenty-first century skills, such as collaboration (Eguchi, 2013), computational thinking (Lee et al., 2011) and problem-solving (Highfield, 2010). Teachers influence the way ER is received by their pupils (Hussain et al., 2006) and therefore play an important role in its implementation in the classroom and integration in the curriculum. Providing teachers with specialized training programs in ER could contribute to ER technologies being introduced into the teaching and learning process. Moreover, student teachers’ responses to ER, such as their perceptions and self-efficacy, could be used to enrich current ER training initiatives.

Pre-service and in-service teachers’ perceptions of ER and the difficulties they encounter in ER classroom implementation are examined in several recent studies. For example, Karypi (2018) put in-service teachers’ perceptions in context by researching their views on ER integration and implementation in schools. Aksu and Durak (2019) also studied in-service teachers’ views on robotics but in the context of robotic tournaments, while Çiftçi et al. (2020) explored pre-service early-childhood teachers’ views on STEM education and their STEM teaching practices. Prior to these studies, Santos et al. (2016) researched in-service teachers’ beliefs, attitudes, and intention to use robotics in their future teaching, while Khanlari (2013) explored in-service teachers' perceptions of the effects of robotics on students’ personal skills and abilities. According to these studies, teachers hold positive views of ER and its impact on students’ learning. Teachers perceive robotics to have positive effects on students' lifelong learning skills (Khanlari, 2016), they consider that most students improve their skills, such as problem-solving, collaboration and creativity, through ER, and acquire engineering and programming skills (Schina et al., 2020; Theodoropoulos et al., 2017). Teachers also perceive that ER promotes students’ curiosity and engages their attention (Aksu & Durak, 2019). In addition, they consider that ER fosters positive attitudes towards STEM education, encourages independent and active learning, facilitates teaching, and provides opportunities for the development of students’ cognitive, social and communication skills (Karypi, 2018). However, there is only limited research on teachers’ self-efficacy towards ER, as current research is mostly focusing on students’ self-efficacy (Durak et al., 2019; Jäggle et al., 2020; Latikka et al., 2019; Leonard et al., 2016; Tsai et al., 2021) rather than the teachers’ self-efficacy. Interestingly Tsai et al. (2021), and Jäggle et al. (2020) propose developing tools for evaluating self-efficacy, for assessing students’ self-efficacy for learning robotics and measuring students’ self-efficacy in educational robotics activities, respectively. Future research should move in this direction, reinforcing teachers’ self-efficacy and measuring it, particularly in ER teacher training programs, in order to improve the structure and content of the training activities.

The present study addresses the need for conducting further research into teachers’ perceptions and self-efficacy towards ER in the context of teacher training. To be more precise, this study examines whether pre-service preschool teachers’ acceptance and self-efficacy towards ER change after they participate in the training program. The study also explores the pre-service teachers’ perceptions of the training program. The research questions are formulated as follows:

- RQ1: To what extent did the ER teacher training program have an effect on pre-service teachers’ acceptance of ER?
- RQ2: To what extent did the ER teacher training program have an effect on pre-service teachers’ self-efficacy in ER?
- RQ3: What are the participants’ perceptions of the ER training program?

By addressing these research questions, the present work contributes to the research and education community in the following three ways:

- The study places pre-service preschool teachers at the center of the ER teacher training research. As it was pointed out in our review (Schina, Esteve-González, et al., 2020), there are very few training programs held exclusively for preschool teachers (Bers et al., 2002, 2013; Caballero-González & Muñoz-Repiso, 2017). This training program is tailored to the specific needs of preschool teachers. Our study therefore addresses a gap in the present literature and could enrich the work of other researchers.
- The study looks at pre-service teachers’ acceptance, self-efficacy, and perceptions throughout an ER training program. These variables are decisive when it comes to teachers’ classroom implementation of ER activities and ER curriculum integration. Our findings could be of use to policy makers who are considering implementing ER teacher training programs.
- The present teacher training program could serve as an example of teacher education that could be replicated at other universities and teacher training institutions. Therefore, it could be of particular interest for institutions and instructors that intend to implement ER teacher training programs.

In the following section, the theoretical framework of our work will be presented in relation to teachers’ ER acceptance and self-efficacy. Then, the methodology of the study will be explained together with the context, population, training description, instruments and data analysis. The findings will be presented in the results section (Sect. 4). Finally, we compare our findings with the research results in the current literature in the discussion and conclusion section (Sect. 5).

## Theoretical framework

In order to promote technology in education, it is recommended that specialized training be implemented. Teachers need to receive training to ensure that they can integrate technology into teaching in meaningful ways to support K-12 student learning (Casey et al., 2020). Effective training in technology integration focuses on content (including technology knowledge and pedagogy-related knowledge and skills), gives teachers opportunities for ‘‘hands-on’’ work, and addresses teachers’ needs (Hew & Brush, 2007). In the case of Digital Technologies (DT), such as robotic kits or robotic toys, apart from knowledge and experience, teachers should have a positive predisposition towards the new resources before teaching the classes in order to transmit positive impressions and enthusiasm to the learners. Hew and Brush (2007) recommend implementing professional development sessions to improve teachers’ perceptions of technological tools. Among teachers’ perceptions, this study will focus on teachers’ acceptance and self-efficacy towards ER as they are both crucial for teachers’ implementation of ER activities in their classroom teaching, and are not sufficiently researched in the current literature as yet.

Regarding teachers’ acceptance of robots in education, Chevalier et al. (2016) point out that teachers’ acceptance depends on the time they need to become acquainted with the robots and the robots’ appropriateness for the curriculum. Chevalier et al. (2016) also highlight that if teachers are provided with more training opportunities and pedagogical materials that can be used directly and are linked to the curriculum, their perceptions of usability of robots improve and therefore their acceptance of robotics in education also increases. Moreover, Conti et al. (2017) suggest that teachers would be more positive and accepting of robots in education if robots were cheaper. Similarly, according to the research of Park and Han (2016), teachers’ acceptance of robot-assisted learning environments mainly depends on the price of the robot. The teachers’ acceptance of robotics and other technological resources can be measured through different research instruments. The Technology Acceptance Model (TAM) (Davis, 1989) has been widely used in educational technology contexts, including ER; for example, it has been used to analyze teachers’ responses to open-ended questions to identify and determine their views regarding the perceived usefulness and perceived ease-of-use of floor-robots as a classroom technological tool (Casey et al., 2020). It has also been used to examine Computer Science teachers’ perceptions, beliefs, and attitudes on Computational Thinking (Fessakis & Prantsoudi, 2019). Subsequent to the Technology Acceptance Model (TAM) and its derived variations, the literature suggests the Unified Theory of Acceptance and Use of Technology (UTAUT) (Venkatesh et al., 2003) to measure teachers’ perceptions because this model integrates the previous TAM variations. Conti et al. (2017) applied the Unified theory of Acceptance and Use of Technology model to study the factors that may influence the teachers’ decision to use a robot as an instrument in their teaching practice. Zacharia et al. (2015) developed the Simulation Acceptance Model (SAM) to address the need for an instrument for researching teachers' beliefs, attitudes, and intentions to use simulations for educational purposes. Santos et al. (2016) used SAM to assess teachers’ beliefs, attitudes, and intentions to use the Lego Mindstorms software in their teaching. Later, Park and Han (2016) developed a variation of the Technology Acceptance Model, called the Robot Service Acceptance Model (RSAM), that is specialized in examining teachers’ views on robot-assisted learning environments with a cloud service platform.

As far as teachers’ self-efficacy is concerned, self-efficacy towards Digital Technologies (DT) and their classroom integration has been an issue of interest for a long time now in the field of education. Russell and Bradley (1997) expressed their concern regarding teachers’ lack of self-efficacy in DT, pointing out that “there is considerable evidence to suggest that schoolteachers in many countries are not confident in the use of computers”. To be more precise, Jones (2004) related this lack of teachers’ self-efficacy to their lack of competence in DT. To achieve high levels of self-efficacy in digital technologies, teachers’ competence should be improved, and this can be achieved through teacher training (Jones, 2004). A limited amount of studies have been carried out on self-efficacy in ER over the last decade (Hamner et al., 2016; Hodges et al., 2016; Jaipal-Jamani & Angeli, 2017; Liu et al., 2010; Santos et al., 2016). Their findings suggest teachers’ self-efficacy for teaching with robotics can be improved with an ER training program (Hamner et al., 2016; Jaipal-Jamani & Angeli, 2017; Liu et al., 2010). Interestingly, Hodges et al. (2016) found that teachers had high levels of self-efficacy towards the implementation of the new problem-based science curriculum throughout the entire professional development program. The results of the previous research are promising regarding the effect of training on teachers’ self-efficacy in ER. However, the studies’ limited sample sizes place in question the reliability and generalizability of their outcomes.

## Methodology

A one-group intervention study (Creswell & Guetterman, 2019) was used as we aimed to examine the relationship of participants’ acceptance and self-efficacy towards ER and the change in their perceptions as a result of an ER teacher training program. The results of this quantitative study can provide insights into other, similar situations and cases and therefore assist in their interpretation (Cohen et al., 2007). The study was conducted using a associational design (Krause, 2018) to collect data. Associational research is appropriate for providing a context for dealing with many variables and studying their relationships and differences. In our study, there were two quantitative instruments (pre-post tests on acceptance and self-efficacy) and one qualitative technique (training journals on perceptions). These were applied in parallel within a short time during one university term. More information on the data collection instruments is provided in Sect. 3.2. (Research Instruments). The quantitative and qualitative results were analyzed separately, and the findings answer different research questions that are interpreted in the conclusions section of this paper (see Sect. 5). Our study measures the impact of an intervention. The ER teacher training program is evaluated in terms of participants’ acceptance and self-efficacy towards ER and participants’ perceptions.

### Context, population and training description

The research was conducted in the framework of the university course entitled “Teaching and Learning of the Experimental, Social and Mathematical Sciences III” part of the degree in Preschool Education at the University of Rovira i Virgili. The university course is addressed to 4th year university students and gives a total amount of 6 ECTS credits. The present research study took place in February, March, and April 2020 in the framework of the research project “INTROBOT” and offered participants a 6-h training program in ER that was both on-site and online.

The population of the research study consisted of 90 pre-service preschool teachers. The average age of the participants was 22.9 (SD = 1.985). All pre-service preschool teachers in our population had previously carried out teaching practice as part of their university studies. The demographic profile of the participants is shown in Table 1. The convenience sample technique was used as it is a fast and economic way of sampling that allows easy access to available participants; however, it does not yield a representative sample of the target population (Cohen et al., 2007).

**Table 1:** Participants’ demographic profile

Participants	People (n)	Percentage (%)
Gender		
Male	5	5.5
Female	85	94.5
Interests		
Participants interested in learning about ER	90	100
Participants interested in learning how to apply ER in their teaching	90	100
Experience		
Participants with prior contact with ER in their personal life	45	50
Participants who used ER resources in educational contexts	47	52

The ER training program consisted of three sessions, the first two sessions took place on the University premises during the last week of February and the first week of March 2020, while the 3rd session took place asynchronously online in the first week of April 2020 due to the COVID-19 pandemic (Table 2). The training was designed based on the constructivist learning approach and project-based learning. In the first session the pre-service teachers were introduced to Educational Robotics. The pre-service teachers were presented to the most widespread educational robotics resources, especially the ones used at a preschool level. In addition, the pre-service teachers were introduced to programming and to concepts such as algorithms, sequencing, debugging, and the instructor presented the definition of Computational Thinking (CT) as stated by Wing (2011). After this brief theoretical introduction, the Blue-bot robotic toy and its functions was presented to the pre-service teachers. They then had the chance to experiment with this resource in groups carrying out several scaffolded programming tasks and debugging challenges set by the instructor. After that, the pre-service teachers experimented with six different Blue-bot classroom projects and materials by carrying out in groups the interdisciplinary activities that addressed socio-economic issues and the protection of the natural habitat. Through these projects, they became familiarized with the interdisciplinary application of the Blue-bot robotic toy in preschool education and with the instructional materials required for implementing it. The second session of the training provided the pre-service teachers with guidelines on Blue-bot robotic toy classroom implementation activities and on the creation of instructional materials. After receiving the guidelines, the pre-service teachers formed groups and were asked to brainstorm on a Blue-bot project for preschool pupils on the following topic: “Vegetation and/or Wildlife in the region of Catalonia in Spain”. For the third training session, the pre-service teachers had to create a project on the above-mentioned topic, including a lesson plan and the teaching materials required for its implementation. In addition, they had to prepare a video presentation of their project in which they presented the learning objectives of their lesson plan, the teaching procedure, a description of the activities and the instructional materials elaborated for the purpose of the given lesson plan. The research team set a month’s interval between the second and the third session so that the pre-service teachers had enough time to work on the Blue-bot project and presentation. The third and last session took place online due to the COVID-19 pandemic. In this session, the pre-service teachers watched asynchronously the other groups’ presentations and evaluated them through an online questionnaire that the research team had elaborated based on evaluation criteria associated with the learning objectives, lesson plan description, teaching materials and a general evaluation. Apart from the other groups’ evaluation, the students had to complete a self-evaluation of their own work. The final grade depended on their 360° evaluation referring to the average of their self-evaluation, peer evaluation and teacher evaluation.

**Table 2:** Training content and research instruments

	Session content	Research Instruments
Session 1 Week 1 (onsite)	• Introduction to Educational Robotics and presentation of ER resources for preschool education • Introduction to Programming and Computational Thinking Experimentation with a Blue-bot robotic toy • Experimentation with Blue-bot classroom projects and teaching materials	1. Q1_pre (quantitative data) 2. Q2_pre (quantitative data) 3. Student Journal Session 1 (qualitative data)
Session 2 Week 2 (onsite)	• Recommendations on Blue-bot robotic toy activities implementation and creation of instructional materials • Students brainstorming on a Blue-bot project for preschool pupils on the topic: “Vegetation and/or Wildlife in the region of Catalonia in Spain”	1. Student Journal Session 2 (qualitative data)
Session 3 Week 6 (online)	• Asynchronous presentation of the Blue-bot projects, asynchronous evaluation of the Blue-bot projects	1. Q1_post (quantitative data) 2. Q2_post (quantitative data) 3. Student Journal Session 3 (qualitative data)

## Research instruments

For the purpose of this research study, the pre-service preschool teachers who participated in the training sessions completed the following questionnaires (see Table 2 above):

- A prequestionnaire (Q1_pre) and postquestionnaire (Q1_post) on the acceptance of ER, quantitative data.
- A prequestionnaire (Q2_pre) and postquestionnaire (Q2_post) on self-efficacy for teaching robotics, quantitative data.
- A journal on their perceptions of the training, qualitative data.

The first questionnaire (Q1_pre and Q1_post) was adapted from the TAM Diagnostic instrument (Davis, 1989) and more precisely from the Spanish version “Instrumento de diagnóstico del TAM” (Cabero & Perez, 2018). It is structured around five sections and uses a 7-point Likert scale ranging from Totally Disagree to Totally Agree. There are 15 items in the questionnaire, which are organized in five dimensions as follows (each dimension is the average of its items, see Table 5 in Appendix): four items on ER usefulness (U1-U4), three items collecting information on ER ease of use (F1–F3), three items on ER enjoyment (D1–D3), three items on attitudes towards ER use (A1–A3), and two items on intention to use it the future (I1–I2). The questionnaire items are provided in Appendix in the original language (Spanish). The main sections of the prequestionnaire (Q1_pre) and postquestionnaire (Q1_post) are exactly the same; however, in the prequestionnaire (Q1_pre) there are some additional demographic questions that collect supplementary information on the research sample. In the second questionnaire (Q2_pre and Q2_post) there are six items that collect information on the self-efficacy of pre-service teachers in relation to their ability to make efficient use of ER in the classroom as a teaching resource (Q1–Q6). This questionnaire was adapted from the Self-efficacy for Teaching Robotics Questionnaire in the research study of Jaipal-Jamani and Angeli (2017) and applies a 5-point Likert scale ranging from Totally Disagree to Totally Agree. The self-efficacy value is the sum of the 6 items (see Table 6 in Appendix). The questionnaire items are provided in Appendix in the original language (Spanish). Finally, to gain an insight into pre-service teachers’ perceptions of the training sessions delivered and their perceptions of ER as a teaching resource in preschool education, the participants were asked to complete a journal after each of the three training sessions following the instructors’ guidelines. The Q1_pre and Q2_pre questionnaires were completed at the beginning of the first training session in week 1. The Q1_post and Q2_post questionnaires were completed at the end of the third training session in week 6 to study whether the training program had had an effect on pre-service teachers’ acceptance and self-efficacy towards ER. The participants were asked to complete their training journals after each session in week 1, week 2 and week 6 because the objective was to collect feedback from the participants on each session held.

### Data analysis

All data from questionnaires were transferred to SPSS 26.0 version and analyzed using descriptive statistics. As explained above, these instruments had been studied in previous research to be valid tools for measuring the desired construct. However, Cronbach Alpha was calculated for each dimension of the two questionnaires, both for pre- and post-questionnaire’s data (Cabero & Ruiz, 2018). Although some researchers admit that arithmetic operations cannot be performed in Likert-scale items, other experts (Jamieson, 2004; Sousa & Rojjanasrirat, 2010) affirm that if there is an adequate sample size (at least 5–10 observations per group) and if the data are normally or nearly normally distributed, parametric statistics can be used with Likert scale ordinal data. Furthermore, Norman (2010) provided evidence that parametric tests can be used with data from Likert scales, and give generally more robust results than nonparametric tests (Sullivan & Artino, 2013). Thus, mean and standard deviation (SD) were used as descriptive statistics, and paired samples t-test was used for comparing pre- and post-test results, the size effect was calculated (see Table 7 in Appendix) as a power analysis as the sample size was close to the minimum (Bujang et al., 2018). We used an enumeration process to carry out a content analysis of the qualitative data collected from pre-service teachers’ journals. The enumeration process counts categories and the frequencies of codes, analysis units, terms, words or ideas (Cohen et al., 2007). The content was analyzed on two levels: descriptive and inferential. Relationships among qualitative data were explored by tabling the frequencies and percentages of occurrences of categories (tabulation) and examining their connections (cross-tabulation). The content analysis was carried out by two coders. The coders decided together the codes to be used in the analysis and constructed the analysis categories. Then, the content was coded, and data were categorized in sequential order. Inconsistencies between the two coders were discussed and a consensus was reached for any differences in categorizing and a 100% unity of agreement was achieved.

## Results

The results are presented in relation to the three research questions.

- RQ1: To what extent did the ER teacher training program have an effect on pre-service teachers’ acceptance of ER?

First, the Cronbach's alpha test was run in order to measure the internal consistency or reliability of the questionnaire on the acceptance of ER (Q1) (Cohen et al., 2007). The test was run twice, once for the prequestionnaire and once for the postquestionnaire. The Cronbach Alpha for each dimension in the pre-test was: α (ER usefulness) = 0.885; α (ER ease of use) = 0.867; α (ER enjoyment) = 0.859 α (attitudes towards ER) = 0.687, and α (intention to use) = 0.889. The total number of items of the prequestionnaire was 0.890. For the postquestionnaire: α (ER usefulness) = 0.890; α (ER ease of use) = 0.798; α (ER enjoyment) = 0.905; α (attitudes towards ER) = 0.631, and α (intention to use) = 962, total was α = 0.911, which indicates a high level of internal consistency, except for the attitudes scale; figures that meet the results of Cabero and Perez (2018) research with a sample of 274 students.

**Fig. 1:**
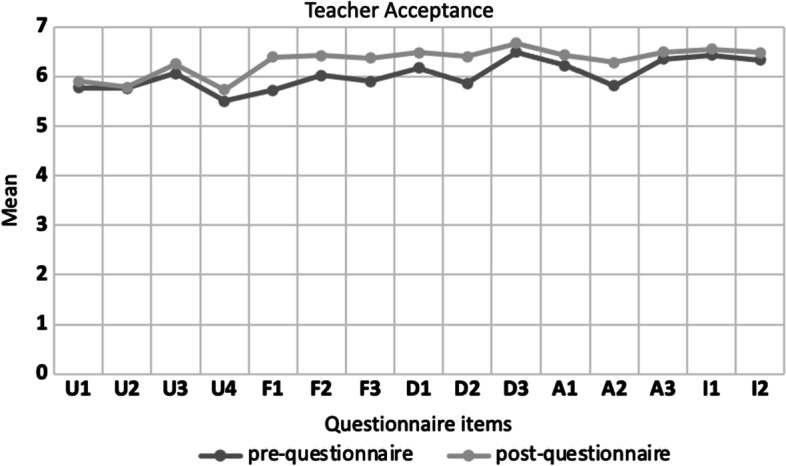
Teachers’ ER acceptance pre/post-questionnaire

Descriptive statistics conducted on the data from the pre-service teachers’ questionnaires showed an improvement in their acceptance of ER after taking part in the ER teacher training based on the data from the prequestionnaire (M = 89.54, SD = 10.28) and postquestionnaire (M = 93.76, SD = 10.07). This questionnaire items are in a 7-point Likert scale and among its items there is a negative-worded item (A2—I feel bored when I use the Blue-Bot). In the data analysis, the scoring scale has been reversed for this specific item. Pre-service teachers’ acceptance improved in all questionnaire items without any exception (Fig. 1). The two-tailed paired-sample t-test showed that there is a statistically significant difference between the prequestionnaire and the postquestionnaire in 7 out of the 15 items (see Table 7 in Appendix). First, the differences between the prequestionnaire and postquestionnaire are statistically significant (95% confidence level) in the section ease of use (F1—It is easy to use the Blue-bot, F2—Learning how to use the Blue-bot wasn’t a problem for me, and F3—Learning how to use the Blue-bot was clear and easy to understand), the effect size for this analysis (d = 0.461) was found to be near to Cohen’s convention for a moderate effect (d = 0.50), suggesting that the training had a relative positive impact on how pre-service teachers perceive how easy it is to use the ER resource. There are also statistically significant (95% confidence level) differences in the sections of enjoyment (D1—Using the Blue-bot is fun, and D2—I enjoyed using the Blue-bot)) with effect size for this analysis (d = 0.412) and attitudes (A1—Using the Blue-bot makes learning more interesting, and A2—I feel bored when I use the Blue-Bot), with also a moderate effect size (d = 0.342).

- Research Question 2: To what extent did the ER teacher training program have an effect on pre-service teachers’ self-efficacy in ER?

First, the Cronbach's alpha test was run to measure the internal consistency or reliability of the questionnaires. The test was run twice, once for the prequestionnaire and once for the postquestionnaire. The Cronbach Alpha for the total number of items of the prequestionnaire was 0.855 and for the postquestionnaire it was 0.873, which indicates in both cases a high level of internal consistency for the self-efficacy scale.

**Fig. 2:**
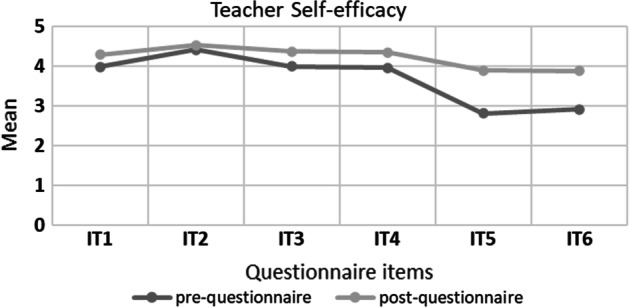
Teachers’ ER self-efficacy pre/post-questionnaire

The results showed an improvement in pre-service teachers’ perceptions after taking part in the ER teacher training. based on the prequestionnaire (M = 22.06, SD = 4.412) and postquestionnaire (M = 25.28, SD = 3.013). This questionnaire is in a 5-point Likert scale. The improvement in pre-service teachers’ self-efficacy was evident in all questionnaire items because all the means of all items increased in the postquestionnaire (Fig. 2). A two-tailed paired-sample t-test at a 95% confidence level showed that there is a statistically significant difference (t(89) = 7.016; p < 0.05) between initial self-efficacy (M = 22.06, SD = 4.412) and final self-efficacy (M = 25.28, SD = 3.013) both measured as the sum of the items of the pre- and the post-test (see Table 8 in Appendix).The effect size (d = 0.740) was found to be close to Cohen’s convention for a high effect (d = 0.80). In particular, statistical differences are observed in the following items: IT1—I feel confident that I have the skills necessary to use robotics for classroom instruction, IT3—I feel confident that I can help my students when they have difficulties with robotics, IT4—I feel confident about teaching students science using educational robots, IT5—I have sufficient knowledge about robotics to integrate it in the learning and teaching process and IT6—I have sufficient knowledge of computational thinking for the development of classroom robotics activities.

- RQ3: What are the participants’ perceptions of the ER training program?

The data collected from pre-service teachers’ journals provide information on their perceptions of each training session (Table 3) and of the entire training program (Table 4) as an overall evaluation of the course.

**Table 3:** Pre-service teachers’ perceptions of training sessions 1, 2 and 3 (F = frequency)

	Codes	Session 1		Session 2		Session 3	
		F	%	F	%	F	%
Session 1 and 2	A. The session was interesting	59	65.6	40	44.4	–	–
	B. The session was useful	37	41.1	45	50	–	–
	C. The session was entertaining	14	15.6	8	8.9	–	–
	D. The session was practical	35	38.9	35	38.9	–	–
Session 2	E. The session supported participants’ collaborative work on the project	–	–	53	58.9	–	–
Session 3	F. The presentation and evaluation of the projects were interesting	–	–	–	–	21	23.3
	G. The presentation and evaluation of the projects were useful	–	–	–	–	27	30
	H. The projects’ online evaluation was practical	–	–	–	–	9	10
	I. The online self and peer evaluations helped us recognize the strong and weak aspects of our project and/or our peers’ projects	–	–	–	–	29	32.2
	J. The self, peer and teacher evaluations were fair	–	–	–	–	19	21.1
	K. I’d prefer to try out the projects in class	–	–	–	–	4	4.4

**Table 4:** Pre-service teachers’ overall perceptions of the training (F = frequency)

Codes	F	%	Codes	F	%
L. The training was innovative	21	23.3	P. Additional ER resources are necessary	14	15.6
M. Participating in the training was useful	48	53.3	Q. Additional time for experimentation with the resources is necessary	31	34.3
N. Participating in the training was entertaining	18	20	R. Preference for completing the training on-site	22	24.4
O. Participating in the training was interesting	46	51.1	S. Additional training sessions are necessary	15	16.7

The following themes emerged from the qualitative data for sessions 1 and 2 based on the pre-service teachers’ perceptions of each training session: the session was considered interesting, useful, entertaining, practical and helpful for participants’ collaborative work on the project (codes A–E, see Table 3). Examining session 1 closer, the most frequent code is A “The session was interesting”, which was counted 59 times in total in 90 journals, while code B “The session was useful” is also very frequent, counted 37 times. A pre-service teacher explained that “during the bachelor’s degree we did not experiment enough with digital technologies in education”. Another pre-service teacher reported that “in the first training session we saw first-hand how to integrate the ER resource into educational contexts”. In session 2, the most frequent code is E “The session supported participants’ collaborative work on the project”, which is found in 53 journals, which is more than half of the total number of journals. A pre-service teacher explained that “the session was very effective as we discussed our thoughts on the project with the rest of the group members and received guidelines and feedback from the trainers”. Code B “The session was useful” is also mentioned very frequently in the journals of session 2 (45 times). In both sessions, code D “The session was practical” is encountered 35 times in the journals. As the content and modality of Session 3 was online due to the COVID-19 pandemic, different codes were selected for the journals’ content analysis: “the presentation and evaluation of the projects were interesting and useful”; “the projects’ online evaluation was practical”; “the online self and peer evaluation helped us recognize the strong and weak aspects of our project and our peers’ projects”; “the self, peer and teacher evaluations were fair”; and “I’d prefer to try out the projects in class” (codes F–K, see Table 3). Code I was the most frequent and was found in 29 journals. A participant explained that “the peer online evaluation enabled our group to observe the aspects that we did not take into account in the creation process of our project”. Interestingly, code H “The projects’ online evaluation was practical” was counted only 9 times, whereas in session 1 and 2 the practicality of the sessions was mentioned 35 times each. By examining this closer, two participants reported that apart from completing the online evaluation rubric, they would like to provide direct feedback to their peers. The fairness of evaluation was also a topic discussed in the journals, with 19 participants stating that the self, peer and teacher evaluations were fair for the project evaluation, while seven participants expressed their concerns regarding the objectivity of the self and peer evaluations.

Examining the results of the qualitative analysis altogether, it was observed that there were considerably less codes counted in journal 3. In the first session the count of the selected codes was 145, in the second session the code count was 186, while in the third session the code count was 105. In line with this, the researchers who performed the content analysis reported that in the third session particularly, the participants’ journals were completed quickly, often without providing thorough answers. The training participants did not seem to be as meticulous as the research team expected in the journal of the third session. Although handing in the journals for each session was compulsory to complete the training, there were 6 pre-service teachers in our sample who did not hand in any of the journals.

The pre-service teachers’ perceptions and overall evaluation of the training program are summarized below in Table 4. The codes L–O refer to positive aspects of the training, while the codes P–S refer to the deficiencies observed by the participants. Regarding the positive aspects, the participants characterize the training as useful (code counted 48 times) and as interesting (code counted 46 times). Presenting their feedback in more detail, many of the participants report that the training was particularly useful for their professional future as teachers and should be part of their teacher education at the university. On the other hand, the deficiencies of the training reported have to do with the integration of additional ER resources in the training, additional time for experimentation with the resources, additional training sessions and the preference for completing the training on-site. The most frequent of all was the need for additional time for experimentation with the resources, counted in 31 journals, while the preference for completing the training on-site was also expressed quite often (22 pre-service teachers stated this).

## Discussion and conclusions

The main aim of the study was to examine whether pre-service preschool teachers’ acceptance and self-efficacy towards ER change after they participate in the teacher training program, together with their perceptions of the training. The results of our study demonstrated an improvement in pre-service teachers’ acceptance of ER after taking part in the ER teacher training, regarding the ease of use of Blue-bot resources, enjoyment and their attitudes towards the Blue-bot resource. In parallel, pre-service teachers’ perceptions of the training collected from the training journals, were very positive. Pre-service teachers viewed the training as innovative, useful, entertaining and interesting, which was in accordance with the quantitative data on their acceptance of ER. Based on our results, pre-service teachers would be eager to accept the Blue-bot in their future teaching in preschool education institutions after engaging in this training. The results of our study go one step further than previous research in the field by offering substantiated quantitative results merged with qualitative data on pre-service teachers’ perceptions. Our study does not aim to obtain any sort of representativeness or generalization, but rather, its goal is to examine the given case. The results of our study complement the previous qualitative results of the research of Casey et al. (2020) who inferred that the 32 pre-service teachers enrolled in an undergraduate education course were positive about the perceived ease of use and usefulness of floor-robots as an educational tool. Our findings confirm the qualitative results of Casey et al. (2020) regarding the improvement in the perceived ease of use of the floor-robots after the course, with significant differences in quantitative data. In relation to the improvement in the teachers’ perception of the usefulness of ER presented in Casey et al. (2020), our study also infers an enhanced perception of usefulness after the training; however, we could not confirm this with significant differences in quantitative data. Nevertheless, the qualitative data collected from the pre-service teachers’ journals depict ER and the ER training as useful for pre-service teachers’ career and professional development and highlight the need for additional ER training sessions and familiarization with more ER resources.

The study results on self-efficacy showed a significant improvement in pre-service teachers’ self-efficacy after taking part in the ER teacher training. The results of our study are in line with those of Jaipal-Jamani and Angeli (2017), whose findings showed that engaging with robotics in a university course can improve pre-service teachers’ self-efficacy for teaching with robotics as well as their computational thinking skills. In addition, our study results are consistent with findings that suggest that teachers improved their self-efficacy for teaching with robotics due to the training received (Hamner et al., 2016; Liu et al., 2010). Although our results cannot be generalized, they enrich the current literature on self-efficacy towards ER, with quantitative data from a broader sample compared to previous research in this discipline. The improvement observed in pre-service teachers’ self-efficacy towards ER could possibly be related to their positive perceptions of ER and of the content and the structure of the training itself.

Nevertheless, although our quantitative data suggest that the participants seem to accept the Blue-bot resource and to improve their self-efficacy towards ER throughout the course, there is no possible way to confirm that pre-service teachers will introduce the Blue-bot or other ER resources in their future teaching contexts. Despite the difficulty of retrieving information on pre-service teachers’ actual classroom ER integration, we consider that the results of this research are very promising as pre-service teachers’ acceptance and self-efficacy seem to improve considerably as the training progresses. We expect that in future ER training programs consisting of additional sessions, involving more resources and more time for experimentation, the improvement in acceptance and self-efficacy will be even more noticeable. Finally, the major contribution of this study is based on the profile of the participants: the study population consists exclusively of pre-school teachers who are rarely included in research on teacher training in educational robotics.

The largest limitation of the study, as mentioned above, is the fact that the pre-service teachers participating in the training did not have the chance to implement the projects they designed in a classroom setting due to the COVID-19 pandemic. The participants did not have the opportunity to apply the knowledge acquired through the training in the preschool educational context because schools in the region remained closed for the rest of the school year after the breakout of the pandemic in March 2020. The general positive feedback received about the content and structure of the ER teacher training program encourages our research team to employ the constructivist learning approach and project-based learning in future training programs. However, in future editions and replications of this training program, we recommend incorporating the following suggestions, some of which were provided by the training participants in their training journals:

- The training program could be extended by adding supplementary training sessions. The extension of the training could have a positive effect on participants’ acceptance, self-efficacy, and perceptions.
- The training program could include familiarization and activities with additional ER and programming resources. For example, it could include sessions with other resources suitable for preschool education, such as Scratch Jr (Papadakis et al., 2016), KIBO (Bers et al., 2019), RoboTito (Gerosa et al., 2019), and Bee-bot (Di Lieto et al., 2017).
- Participants should be given more time for experimentation with the ER resources and ER teaching materials. This would enable the participants to feel more comfortable and confident with the resources and enjoy the learning process without feeling that they need to hurry, which would possibly have a positive effect on their acceptance, self-efficacy, and perceptions.
- In terms of the research design, pre-service teachers’ journals should be improved to provide richer qualitative results. That the frequency of codes counted dropped in the third session, suggests that the students completed the third journal in a rush without spending time on this task. Therefore, in future implementations of this study we recommend dedicating classroom time to this task or in the case of an online modality, set the deadline for handing in the journals shortly after the session.
- In future implementations of this study, a larger sample should be included to increase the reliability of all the instruments and scales, having a sample greater than 300 could allow us to confirm and generalize the positive results and conduct a structural equation model study.
- Future ER training programs could be conducted entirely or partly online in health emergencies like the COVID-19 pandemic. Online and blended versions of the training could incorporate additional features, such as immediate feedback to the students on robotics tasks and/or teaching material creation and online robotics simulations. The pre-service teacher education course presented in Moorhouse (2020) provides insights into the adaptations needed for an online course. The adaptations include making the VCS sessions obligatory, using small group discussions (breakout rooms), reinforcing the structure of the sessions, adding a preparation task with the session materials prior to the class, providing time for group discussion and feedback, recording the sessions, and combining synchronous and asynchronous teaching. As Sun et al. (2020) suggested, the universities should view the COVID-19 pandemic as an opportunity to reform the online education they offer by improving the course content, the digital technology employed and management. Therefore, in our context, the COVID-19 pandemic gives our university a chance to make reforms and rethink the content of teacher online education and the digital technology taught.

Apart from the recommendations provided above, the university teaching committee should reflect on the importance of ER in preschool teachers’ education and apply necessary reforms to integrate ER training into the teacher education curriculum.

## Data Availability

The datasets used and/or analyzed during the current study are available in the Zenodo repository https://doi.org/10.5281/zenodo.4553489.

## Appendix

See Tables 5, 6, 7, 8 and 9.

**Table 5:** Items of questionnaire 1

Item ID	Acceptance of ER—questionnaire items
U1	El uso de la BlueBot mejorará mi aprendizaje y rendimiento en esta asignatura
U2	El uso de BlueBot durante las clases me facilitaría la comprensión de ciertos conceptos
U3	Creo que la BlueBot es útil cuando se está aprendiendo
U4	Con el uso de la la BlueBot aumentaría mi rendimiento
F1	Creo que la BlueBot es fácil de usar
F2	Aprender a usar la BlueBot no es un problema para mí
F3	Aprender a usar la BlueBot es claro y comprensible
D1	Utilizar la BlueBot es divertido
D2	Disfruté con el uso de la BlueBot
D3	Creo que la BlueBot permite aprender jugando
A1	El uso de la BlueBot hace que el aprendizaje sea más interesante
A2	Me he aburrido utilizando la BlueBot
A3	Creo que el uso de la BlueBot en el aula es una buena idea
I1	Me gustaría utilizar en el futuro la BlueBot si tuviera oportunidad
I2	Me gustaría utilizar la BlueBot en la enseñanza de varias disciplinas

**Table 6:** Items of questionnaire 2

Item ID	Self-efficacy—questionnaire items
IT1	Considero que tengo las habilidades necesarias para usar la robótica en el aula
IT2	Estoy seguro de que puedo involucrar a mis alumnos para que participen en proyectos basados en robótica
IT3	Estoy seguro de que puedo ayudar a mis alumnos cuando tienen dificultades con la robótica
IT4	Me siento capaz de enseñar al alumnado temas relacionados de ciencias utilizando robots educativos
IT5	Tengo suficiente conocimiento de robótica para integrarla en los procesos de enseñanza-aprendizaje
IT6	Tengo suficiente conocimiento de pensamiento computacional respecto al desarrollo de actividades de robótica en contextos educativos

**Table 7:** Results of questionnaire 1

Item	Mean (SD)/Item—ER perceptions—prequestionnaire	Mean (SD)/item—ER perceptions—postquestionnaire
U1	5.78 (1.130)	5.91 (1.205)
U2	5.77 (1.112)	5.79 (1.353)
U3	6.07 (1.003)	6.26 (0.978)
U4	5.51 (1.229)	5.74 (1.354)
F1	5.73 (1.159)	6.40 (0.818)
F2	6.03 (1.194)	6.43 (1.039)
F3	5.91 (1.088)	6.38 (0.907)
D1	6.18 (1.087)	6.49 (0.811)
D2	5.87 (1.144)	6.41 (1.037)
D3	6.50 (0.783)	6.68 (0.684)
A1	6.23 (0.937)	6.44 (0.836)
A2	5.82 (1.680)	6.29 (1.392)
A3	6.36 (0.839)	6.50 (0.707)
I1	6.44 (0.795)	6.56 (0.751)
I2	6.34 (0.781)	6.49 (0.782)

Mean (SD)		*t*	*p*	Cohen’s d
Usability—pre 5.781 (0.967)	Usability—post 5.925 (1.118)	1.279	0.204	0.135
Ease of Use—pre 5.893 (1.020)	Ease of Use—post 6.404 (0.781)	4.369	< 0.05	0.461
Enjoyment—pre 6.181 (0.898)	Enjoyment—post 6.526 (0.785)	3.244	< 0.05	0.412
Attitudes—pre 6.137 (0.897)	Attitudes—post 6.411 (0.776)	2.957	< 0.05	0.342
Intention of Use—pre 6.394 (0.748)	Intention of Use—post 6.522 (0.753)	1.314	0.192	0.138

**Table 8:** Results of questionnaire 2

Item	Mean (SD)/Item—ER Self-efficacy—prequestionnaire	Mean (SD)/Item—ER Self-efficacy—postquestionnaire
IT1	3.98 (1.016)	4.28 (0.600)
IT2	4.41 (0.652)	4.52 (0.565)
IT3	3.99 (0.977)	4.37 (0.626)
IT4	3.96 (0.982)	4.34 (0.673)
IT5	2.81 (1.027)	3.89 (0.678)
IT6	2.91 (1.077)	3.88 (0.700)
Self-efficacy	22.06 (4.41)	25.28 (3.01)

**Table 9:** Examples of coded journals from session 3 (week 6)

Code ID	Codes	Example comments student 1	Example comments student 2
F	The presentation and evaluation of the projects was interesting	–	–
G	The presentation and evaluation of the projects was useful	–	–
H	The projects’ online evaluation was practical	–	–
I	The online self and peer evaluations helped us recognize the strong and weak aspects of our project and/or our peers’ projects	Pienso que es muy adecuada para saber reconocer los errores y mejorar como futura docente	Hemos podido añadir nuestro punto de vista sobre la creación de las alfombras de diferentes grupos y de la propia, y nos ha servido para darnos cuenta de qué aspectos podríamos mejorar para la próxima vez
J	The self, peer and teacher evaluations were fair	Me ha parecido muy correcta, para identificar los propios errores y ser capaz de evaluar objetivamente a los compañeros de clase	De esta manera, le daremos un enfoque más objetivo a la evaluación y nos facilita aportar nuestra opinión sobre las debilidades y fortalezas de cada grupo y su trabajo realizado
K	I’d prefer to try out the projects in class	–	–
L	The training was innovative	–	–
M	Participating in the training was useful	me ha parecido muy útil para la formación docente y lo encuentro necesario para poder innovar en la educación y mejorar	El hecho de crear una alfombra por grupos me ha parecido una idea genial, porque nos será útil en un futuro si lo queremos poner en uso con nuestros alumnos, y es una manera de probar si la realización de nuestro proyecto sería factible
N	Participating in the training was entertaining	–	
O	Participating in the training was interesting	–	El proyecto de robótica me ha parecido muy interesante para nosotros, los futuros maestros
P	Additional ER resources are necessary	Tener más tiempo para experimentar con las tabletas y diferentes robots, no solo el Blue-Bot, pero por el corto periodo que la hemos impartido me ha parecido muy completa para iniciarte en este mundo	–
Q	Additional time for experimentation with the resources is necessary	Tener más tiempo para experimentar con las tabletas y diferentes robots, no solo el Blue-Bot, pero por el corto periodo que la hemos impartido me ha parecido muy completa para iniciarte en este mundo	–
R	Preference for completing the training on-site	–	–
S	Additional training sessions are necessary	–	–

## Abbreviations

ER: Educational robotics
DT: Digital technologies
TAM: Technology acceptance model
UTAUT: Unified theory of acceptance and use of technology
SAM: Simulation acceptance model
RSAM: Robot service acceptance model
CT: Computational thinking
